# RETRACTION: Effect of Percutaneous Endoscopic Gastrostomy Prior to Oesophageal Cancer Surgery on Postoperative Wound Complications in Patients: A Meta‐Analysis

**DOI:** 10.1111/iwj.70365

**Published:** 2025-03-17

## Abstract

**RETRACTION:** HuangX. , JiaC. , and ZhuY. , “Effect of Percutaneous Endoscopic Gastrostomy Prior to Oesophageal Cancer Surgery on Postoperative Wound Complications in Patients: A Meta‐Analysis,” International Wound Journal 21, no. 2 (2024): e14461, 10.1111/iwj.14461.PMC1082851737905678

The above article, published online on 31 October 2023, in Wiley Online Library (http://onlinelibrary.wiley.com/), has been retracted by agreement between the journal Editor in Chief, Professor Keith Harding; and John Wiley & Sons Ltd. Following an investigation by the publisher, all parties have concluded that this article was accepted solely on the basis of a compromised peer review process. The editors have therefore decided to retract the article. The authors did not respond to our notice regarding the retraction.

**RETRACTION:** X. Huang , C. Jia , and Y. Zhu , “Effect of Percutaneous Endoscopic Gastrostomy Prior to Oesophageal Cancer Surgery on Postoperative Wound Complications in Patients: A Meta‐Analysis,” International Wound Journal 21, no. 2 (2024): e14461, 10.1111/iwj.14461.PMC1082851737905678

The above article, published online on 31 October 2023, in Wiley Online Library (http://onlinelibrary.wiley.com/), has been retracted by agreement between the journal Editor in Chief, Professor Keith Harding; and John Wiley & Sons Ltd. Following an investigation by the publisher, all parties have concluded that this article was accepted solely on the basis of a compromised peer review process. The editors have therefore decided to retract the article. The authors did not respond to our notice regarding the retraction.

